# Phenotypic and Dermoscopic Patterns of Familial Melanocytic Lesions: A Pilot Study in a Third-Level Center

**DOI:** 10.3390/cancers15153772

**Published:** 2023-07-25

**Authors:** Gabriele Roccuzzo, Silvia Giordano, Thomas Granato, Francesco Cavallo, Luca Mastorino, Gianluca Avallone, Barbara Pasini, Pietro Quaglino, Simone Ribero

**Affiliations:** 1 Department of Medical Sciences, Section of Dermatology, University of Turin, 10126 Turin, Italy; 2 Medical Genetics Unit, AOU ‘Città Della Salute e Della Scienza’-‘Molinette’ Hospital, 10126 Turin, Italy; barbara.pasini@unito.it

**Keywords:** melanoma, dermoscopy, familial melanoma, CDKN2A, MITF, OCA1, OCA2, OCA4, TYR, PTEN, SLC45A2, genetics

## Abstract

**Simple Summary:**

The research aims to investigate familial melanoma, a form of skin cancer that has a genetic predisposition. Melanoma is a dangerous cancer with a high potential for metastasis, and early detection is crucial for reducing its impact on patients. The study focuses on identifying specific genetic mutations associated with familial melanoma and correlating them with distinct dermoscopic patterns. By understanding the relationship between genetic mutations and dermoscopic features, the researchers aim to develop reference models to aid clinicians in identifying high-risk patients and their families. Establishing these correlations could lead to improved screening and a timely detection of new tumors in individuals with a family history of melanoma.

**Abstract:**

Cutaneous melanoma is a highly aggressive skin cancer. It is estimated that 5% to 10% of the underlying mutations are hereditary and responsible for familial (or hereditary) melanoma. These patients are prone to the early development and higher risk of multiple melanomas. In recent years, an increasing number of genes have been identified thanks to genetic testing, allowing the subsequent surveillance of individuals at risk, yet it is still difficult to predict the presence of these mutations on a clinical basis. In this scenario, specific phenotypic and dermoscopic features could help clinicians in their identification. The aim of this work has been to correlate mutations to prevalent dermoscopic patterns, paving the way for reference models useful in clinical practice. In our cohort, out of 115 patients referred to genetic counseling for melanoma, 25 tested positive (21.7%) for critical mutations: CDKN2A (n = 12), MITF (n = 3), BAP1 (n = 1), MC1R (n = 3), PTEN (n = 1), TYR (n = 2), OCA2 (n = 1), and SLC45A2 (n = 2). The phenotype profiles obtained through the digital acquisition, analysis, and description of both benign and malignant pigmented lesions showed a predominance of the type II skin phenotype, with an elevated mean total nevus number (182 moles, range 75–390). As for dermoscopic features, specific mutation-related patterns were described in terms of pigmentation, areas of regression, and vascular structures. Although further studies with larger cohorts are needed, our work represents the beginning of a new approach to the study and diagnosis of familial melanoma, underlining the importance of clinical and dermoscopic patterns, which may constitute a reference model for each gene, enabling comparison.

## 1. Introduction

Melanoma is a malignant neoplasm that originates from melanocytes. It mainly localizes to the skin but can also develop, albeit more rarely, in other body districts where melanocytes are present, such as the mucous membranes, uvea, and meninges [1]. It accounts for 5% of skin malignancies, and although less common than other skin neoplasms, such as basal cell carcinoma (BCC) and squamous cell carcinoma (SCC), it is more life-threatening because of its metastatic potential; hence, the early detection and assessment of its progression are critical measures to reduce morbidity [2]. It predominantly affects individuals between the ages of 30 and 60, with a predominance for males [3,4]. About 85% of skin melanomas that occur each year affect populations in North America, Europe, and Oceania [5]. Currently, an estimated 132,000 new cases occur each year worldwide, along with approximately 58,000 deaths [6,7,8]. Concurrent with rising incidence trends, the mortality rate in most of the world has been rising, yet the therapeutic landscape of unresectable stage III and IV melanoma has been recently revolutionized by immunotherapies and targeted therapies, which have markedly improved survival, compared to traditional chemotherapy regimens [9,10,11]. Similar to other cancers, melanoma has a multifactorial etiology, as its genesis results from the association of intrinsic and extrinsic risk factors [12,13]. Intrinsic (or constitutional) risk factors are represented by positive personal and/or family history, immunodepression, race, phototype, the presence of numerous and atypical nevi, and genetic mutations which are responsible for familial clusters of melanomas. As for the extrinsic ones, the most important risk factor is the exposure to natural or artificial sources of ultraviolet radiation because of their genotoxic effect [14,15,16]. Although the definitive diagnosis of cutaneous melanoma is based on histopathologic criteria, the first approach is based on dermoscopic evaluation, which significantly increases diagnostic accuracy by 15–20% compared to clinical observation alone [17,18,19,20].

### 1.1. Familial Melanoma

About 5% to 10% of melanoma cases occur in patients with a family history of melanoma [21]. The early development and higher risk of multiple melanomas have been shown in these patients, and the risk of melanoma increases proportionally with the number of family members affected [22,23]. Moreover, these patients and their families may develop other internal organ malignancies, configuring the so-called mixed cancer syndrome (MCS), for which there is a high incidence of various cancers in general, including melanoma [24]. Although several predisposing genes have been identified over the years, such as CDKN2A (cyclin-dependent kinase inhibitor 2A), MC1R (Melanocortin 1 Receptor), MITF (melanocyte-inducing transcription factor), and BAP1 (BRCA1 associated protein 1), not all cases of familial melanoma can be simply explained by pathogenic germline mutations [25]. A genetical phenomenon known as epistasis, defined as the interaction between two genes where the effect of a particular gene depends on the presence of another modifying gene, has been shown to play a role in the pathogenesis of some familial melanomas [25,26,27]. The CDKN2A cluster of patients was the first to be associated with a higher risk of developing familial melanomas, as this mutation can be found in up to 25% of Caucasian families with multiple melanomas [28]. This gene exerts its function by encoding two tumor suppressors, known as p16 and p14, which act as proliferation inhibitors and apoptosis regulators throughout the cell cycle [29]. The overall risk of developing melanoma in an 80-year-old individual with a family mutation of CDKN2A has been found to be increased by 58% in Europe, 76% in the US, and 91% in Australia [30,31,32]. As for MC1R, this gene is recognized as a main player in human pigmentation, as it binds to the α-melanocyte-stimulating hormone (α-MSH), favoring eumelanin production over pheomelanin [33]. Specific polymorphisms in the MC1R locus have been related to the “red hair color” (RHC) phenotype and impaired DNA repair mechanisms, leading to an increased susceptibility to UV radiation [34,35]. The risk of developing melanoma appears to be higher as the number of MC1R variants increases in the carrier, doubling in carriers of a single variant and rising to six times in those with two or more variants [36,37,38,39]. Another gene involved in cell pigmentation is the melanocyte-inducing transcription factor (MITF) and its germline variant p.E318K, which has been related to a higher risk (i.e., up to five times) of developing melanomas in the carriers [40,41,42]. As for the BRCA1 associated protein 1 (BAP1), it plays a crucial role in cell division and DNA repair, with reported mutations in around 3% of uveal melanomas, especially in patients with metastases [43,44]. Remarkably, around one third of families with both cases of cutaneous and uveal melanomas display mutations in BAP1, as opposed to families with multiple cutaneous melanomas alone, in which they are detected in less than 1% of the cases [43]. Overall, the risk of developing melanoma in individuals with a predisposing mutation appears to correlate not only with the type of mutation, but also with some environmental factors and other unknown variables, thus suggesting a polygenic inheritance mechanism [45,46].

### 1.2. Dermoscopy in Familial Melanoma

Since its introduction in clinical practice, dermoscopy has represented a breakthrough in the world of dermato-oncology, dramatically improving the diagnostic accuracy of melanocytic lesions [1,6,25]. Together with total body photography, it currently represents the primary tool for the diagnosis of melanoma, thanks to its high specificity and sensitivity, especially in high-risk patients with a family history positive for melanoma [1,6,25]. Moreover, genetic counseling with the possibility of testing for predisposing genes represents a further valuable screening tool for these patients [47,48]. As described in the literature, each predisposing gene presents a different intrinsic mechanism leading to the inactivation of fundamental biological mechanisms regulating cellular integrity [25]. These genes can influence the phenotype of several clinical traits, such as the fair skin phototype, with subsequent increased susceptibility to sun exposure. In this scenario, a high nevus count, large congenital nevi, and atypical melanocytic lesions can represent common features in this subset of patients. Therefore, just as the presence or absence of peculiar dermoscopic structures can predict the melanoma subtype (e.g., melanomas on sun-damaged skin often reveal the same dermoscopic pattern), similarly, a specific mutation could potentially influence the phenotypic appearance of melanocytic lesions [25,49]. Unfortunately, despite few preliminary findings, a consensus regarding the correlation between mutations and respective clinical and dermoscopic features is still lacking [25]. In this context, establishing these correlations would help oncologists and dermatologists to enroll predisposed subjects and their families into specific follow-up and screening programs for the timely detection of new tumors.

### 1.3. Objectives

The aim of this work has been to focus on the clinical and dermoscopic patterns of familial melanomas and phenotype patients who tested positive for predisposing genetic mutations, with an attempt to correlate specific mutations to prevalent dermoscopic patterns, paving the way for reference models useful in clinical practice.

## 2. Materials and Methods

### 2.1. Study Crireria

This study was approved by Comitato Etico Interaziendale AOU Città della Salute e della Scienza di Torino (TESEO-0061280) and was conducted in accordance with the principles of the declaration of Helsinki. The Genetics of Familial Melanoma Clinic was established at the University of Turin (Turin, Italy) in 2018 to study and optimize the prevention of hereditary melanomas. According to the 2020 Italian Association of Medical oncology (AIOM) guidelines [6], genetic blood testing for the evaluation of the carrier status for genetic variants associated with familial melanoma has been offered to patients presenting one of the following criteria: patients diagnosed with melanoma with a family history positive for melanoma (at least two family members affected, with at least one case diagnosed within 60 years of age); patients diagnosed with multiple melanomas younger than 60 years of age; and patients diagnosed with melanoma with a family history positive for another tumor among pancreatic adenocarcinoma, uveal melanoma, mesothelioma, renal tumor, and atypical Spitz tumor [50].

### 2.2. Genomic Analysis

The examination of predisposition genes, particularly the search for point mutations, has been performed by next-generation sequencing (NGS), applied to the analysis of the coding region ± 20 intronic bases flanking the exons of the following genes: ACD (adrenocortical dysplasia homologue), BAP1 (Breast Cancer gene 1 Associated Protein 1), CDKN2A (Cyclin Dependent Kinase Inhibitor 2A, exons 1 alpha, 2 and 3), CDK4 (Cyclin Dependent Kinase 4, exon 2), MC1R (Melanocortin 1 Receptor), MITF (Microphthalmia-associated transcription factor, exon 9), POT1 (Protection of telomeres protein 1), TERF2IP (Telomeric repeat-binding factor 2-interacting protein 1), and TERT (Telomerase reverse transcriptase [30,31]. The library was obtained with SureSelect-Agilent Custom Hereditary Cancer Solution probes^TM^ (Agilent, Santa Clara, CA, USA), the sequencing with MiSeq platform Illumina^TM^ (Illumina, Meinz, Germany) and the sequence alignment and variant designation with SOPHiA Genetics DDM software^TM^ version 5.0.13 (Sophia Genetics, Rolle, Switzerland). Along with genetic testing, the patients’ dermoscopic images of melanocytic lesions were acquired digitally through HD medicam 1000s FotoFinder^TM^ (FotoFinder Systems GmbH, Bad Birnbach, Germany) and then analyzed.

### 2.3. Patient Examination

A review of the scientific literature was performed regarding familial melanoma, in particular the correlation between dermoscopic features and mutations. For each gene, based on the findings available, an attempt was made to draw up a dermoscopic model that could be taken as a reference. The clinical and dermoscopic features of melanocytic lesions were therefore described and compared to the currently available literature evidence. Concordance and discordance with the literature data were recorded. Specifically, for each patient, the total nevus number (TNN) was assessed through total body clinical and dermoscopic examination. All parts of the body, including the genital area, scalp, and mucosa (ocular and oral), were analyzed. The examination was manually performed by one consultant (SR) and two resident doctors with more than 2 years of training in dermoscopy. The specific dermoscopic criteria for benign melanocytic lesions were considered, including pattern (reticular, globular, homogeneous, or starburst), pigmentation, regular globules and spots, symmetrical peripheral striae, central blotches, and vascular pattern in the absence of criteria for atypical nevi and melanoma (i.e., irregular spots and globules, asymmetrical peripheral striae, eccentric blotches, blue-white veil, areas of regression, polymorphic vascular structures) [49]. Table 1 summarizes the current evidence available in the literature regarding gene mutations and clinical-dermoscopic features.

## 3. Results

Participation in genetic testing was solely on a voluntary basis and all NGS analyses were performed on a peripherally collected venous blood sample. A total of 115 patients met the inclusion criteria according to the AIOM guidelines and agreed to receive genetic testing [50]. Among them, 25 tested positive for a genetic mutation (21.7%), while the other 90 (78.3%) did not report any specific germline mutation. Table 2 represents the results of genetic testing in our cohort of patients.

Regarding the cohort of patients who tested positive, a slight female prevalence (64%) was observed, with a mean age of 50.6 (range 24–80). All patients (100%) were of Caucasian heritage, with different skin phenotypes as follows: I (10%), II (45%), III (35%), and IV (10%). No type V and VI phenotypes were observed. All patients had a positive history for either (I) a prior melanoma and an affected familial member, or (II) personal multiple melanomas. All patients were referred to our service due to a concomitant new diagnosis of melanoma, with an average number of prior melanomas of 3 (range 1–9). As for the nevi count, a mean TNN of 182 moles (range 75–390) was recorded. As for the body sites of melanomas, the back was the most common one, followed by the chest and upper limbs. No localization on the head/neck, lower limbs, or genitalia were recorded. No acral melanomas were diagnosed either. For each cluster of mutation, the main dermoscopic features of both nevi and melanoma were recorded. Table 3 depicts the patients’ characteristics.

**Figure 1:**
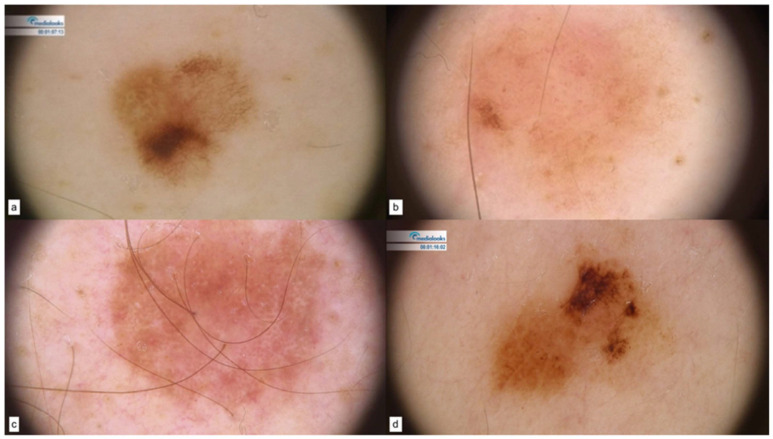
CDKN2A patterns of nevi (**a**,**b**) and melanomas in situ (**c**,**d**).

As for CDKN2A patients, nevi dermoscopy showed the prevalence of a pigmented reticular pattern (Figure 1a), along with hypopigmented lesions (Figure 1b). Melanomas displayed unstructured areas, blotches, atypical network, pinkish areas, and atypical vascular structures. Figure 1c,d display two in situ melanomas.

**Figure 2:**
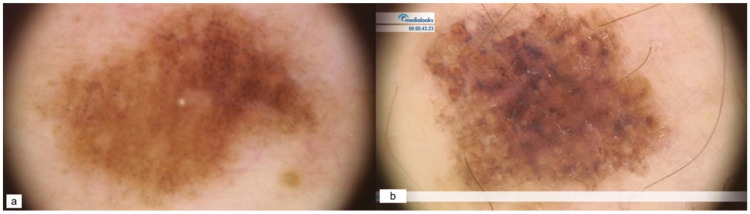
MITF patterns of nevus (**a**) and melanoma (**b**).

Regarding MITF patients, nevi dermoscopy was characterized by pigmented reticular nevi (Figure 2a) and malignant lesions showing non-specific patterns. Figure 2b depicts a superficial spreading melanoma (SSM), Breslow 0.7 mm.

**Figure 3:**
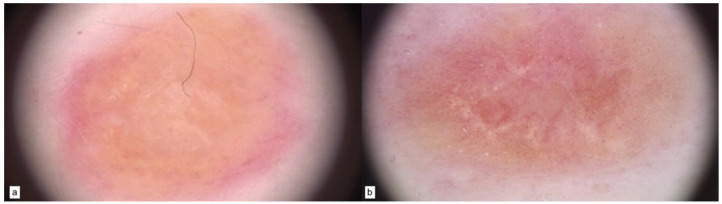
BAP patterns of nevus (**a**) and melanoma (**b**).

Hypomelanotic/amelanotic patterns were the most common features of both benign and malignant melanocytic lesions in our BAP-mutated patient (Figure 3a), with the addition of structureless pink-to-tan and atypical vascular structures in the latter (Figure 3b, SSM, Breslow 0.4 mm).

**Figure 4:**
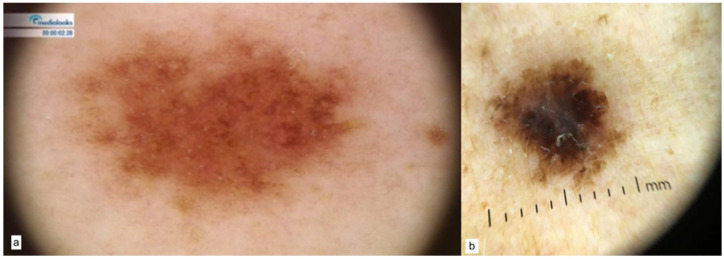
MC1R and PTEN patterns of nevi (**a**) and melanoma (**b**).

As for MC1R- and PTEN-mutated patients, a pigmented reticular pattern was the main dermoscopic feature (Figure 4a), with an atypical network characterizing malignant melanoma (Figure 4b, SSM, Breslow 0.8 mm).

**Figure 5:**
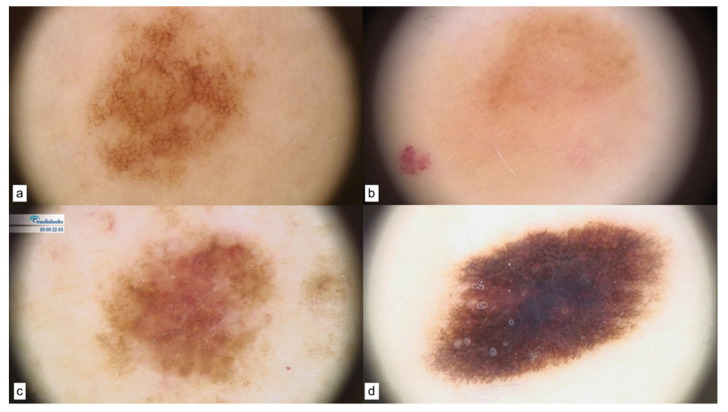
OCA associated patterns of nevi (**a**,**b**) and melanoma (**c**,**d**).

At last, as for the OCA (OCA1, OCA2, OCA4) variants, nevi dermoscopy was characterized by the coexistence of reticular and globular patterns with hypopigmented lesions (Figure 5a,b). The atypical network and hypopigmented areas were seen in melanoma lesions (Figure 5c, MIS, Figure 5d SSM, Breslow 0.5 mm).

## 4. Discussion

In recent years, several studies have been carried out in an attempt to relate the genetic mutations and clinical/dermoscopic features of both benign and malignant melanocytic lesions [65,66,67,68,69,70,71]. However, due to the shortage of registries collecting cohorts of patients with familial melanoma, clear data are still lacking. The aim of this work has been to describe the experience of a tertiary melanoma clinic and compare these preliminary results with the data available in the literature. According to our findings, some interesting observations can be made. First, we confirm that CDKN2A does represent the most common germline mutation in our familial melanoma cohort, as reported in the other cohorts of patients from different Italian regions [72]. According to Bruno et al., the CDKN2A germline mutation is found in around 36.6–58.8% of familial multiple primary melanoma cases, whereas in sporadic multiple primary melanoma, the cases vary from 8.2% to 17.6% [72]. In our cohort, CDKN2A accounted for 48% of the detected germline mutations, followed by MITF (12%) as the second most common one. As for dermoscopic and clinical findings, similarities have been recorded in CDKN2A and MITF patients. In the former, low pigmentation with non-homogeneous coloring is the main pattern in malignant lesions, with pinkish and whitish areas of regression, as previously described [54,62,73,74]. The tendency to develop multiple melanomas at an early age is another clinical feature that CDKN2A patients share. An additional trait that would seem to be characteristic is the development of atypical nevi and a personal history of non-melanoma skin cancers [75,76,77]. Similar evidence can be found in MITF-mutated patients, as they display an elevated TNN with a predominant location at the trunk, as described by Sturm et al., who also report a dominant reticular dermoscopic signature pattern of nevi [56]. As for melanoma, the data from the literature vary. While Sturm et al. describe a high incidence of amelanotic melanomas within the group, Bassoli et al. describe a multicomponent pattern with areas of different pigmentation [56,78]. The latter agrees with our evidence in which an atypical reticular and multicomponent pattern with pinkish and regression areas can be highlighted. Polymorphic vascular structures that are dotted or comma-like can also be found [79,80]. Secondly, BAP1-mutated patients have been reported to develop BIMTs (multiple BAP1-inactivated melanocytic tumors), also known as BAP1-inactivated melanocytic tumors, which are multiple, skin-colored or reddish-brown, dome-shaped melanocytic tumors which tend to develop early in life (median 31 years, range 10–56 years) and increase in number with age [59]. Our results confirm this evidence, as a patient in our study aged 34 had already been diagnosed with two melanomas, a BAPoma, and an atypical Spitzoid tumour (AST). The melanoma dermoscopic features encompass a typical unstructured lesion, pink-to-light brown in color, with eccentrically localized irregular dots or globules, and our patient shared these findings with the cases reported in the literature [81]. As for MC1R, it is known that the presence of loss-of-function in these variants correlates with the red hair color (RHC) phenotype, characterized by fair skin, red hair, the presence of freckles, high sun sensitivity, and a poor tanning response [62,66,67,68,70,71,72]. MC1R variants modulate color (hypopigmentation) and dermoscopic patterns (lack of structures) in nevi and melanomas, resulting in more visible vessels, supporting the theory that *MC1R* is crucial for melanocyte proliferation, regulation, and differentiation, having a growth-promoting effect on melanocytes [55,63,82,83,84,85,86]. Our patients, according to this evidence, developed pigmented melanomas on the trunk in a background of hypomelanotic nevi localized throughout the skin. At last, the literature on OCA genes mainly describes a tendency to develop amelanotic and hypomelanotic melanomas for the carriers of this mutation, highlighting the role of these genes in melanocyte activity and pigmentation. In our experience, the homogeneous structureless pattern, globular pattern, reticular pattern, and peripheral reticular pattern with central hypopigmentation and peripheral globules were the most common in nevi, in accordance with the literature evidence [67,71,87]. Our OCA2-mutated patient developed three melanomas, which showed an atypical pattern with irregular polychromatic pigmentation, dark areas alternating with lighter areas, and pinkish and regression areas. As for TYR (OCA1) and SLC45A2 (OCA4), patients with these mutations showed an irregular pigmentation as the most characteristic dermoscopic aspect of melanomas, despite the common tendency to develop hypomelanotic/amelanotic lesions, as previously reported [67]. As for the latter cluster, our patients developed melanomas with atypical pigmented networks and streaks, despite the absence of concomitant MITF expression, as described by Ozaki et al. [68].

## 5. Conclusions

While familial melanoma remains a rare disease, its impact can be significant, especially in cases with multiple occurrences. Early diagnosis remains crucial, and the identification of predisposing genetic mutations can greatly assist physicians in managing patients with a personal or family history of melanoma. Preliminary data indicate that carriers of certain germline mutations may exhibit specific phenotypic traits, including unique dermoscopic features. Thoroughly understanding the correlation between genotypes and phenotypes on a larger scale could revolutionize the approach to studying and diagnosing familial melanoma. Our study has provided valuable insights, aligning with known patterns, and offered new perspectives on less explored mutations, like the OCA gene cluster. To further advance research, international collaboration is essential due to the rarity of familial melanoma. The creation of an online atlas, based on national registries collecting clinical and dermoscopic data, would equip dermatologists and oncologists with valuable tools to identify melanoma-prone families effectively. Although it is still in early stages, our work strives to contribute to improved diagnostic strategies for this challenging disease.

## Figures and Tables

**Table 1:** Relationship between gene mutation and clinical-dermoscopic features.

Mutation	Frequency [25]	Function [25]	Clinical Features [25,50,51,52,53,54,55,56]	Dermoscopic Features [25,50,51,52,53,54,55,56]
**CDKN2A**	20–40% of FM	Tumor suppressor	-Minor age at melanoma diagnosis [25] -Multiple melanomas [51] -Tendency to SSM in absence of sunburns [52] -Low proportion of acral-nodular melanoma [53] -High TNN, atypical and increasing size nevi [54]	-Unstructured areas (MC1R variants) [55] -Streaks and pigmented network [55]
**MITF**	1–5% of FM	Regulates melanocyte development	-Minor age at melanoma diagnosis [25] -High TNN [56] -Non-blue eye color [41] -Amelanotic and nodular melanomas (back, leg, arm, abdomen) [42,57]	-Reticular pattern in nevi [56] -Three patterns in dysplastic nevi and melanomas [57]: **I** (non-specific) **II** (globular-homogeneous) **III** (reticular-homogeneous)
**BAP1**	<1% of FM	Tumor suppressor	-Uveal melanoma at younger age [58] -Multiple BAP1-inactivated melanocytic tumors (BIMTs): nodular features [25,59]	Five dermoscopic patterns [25,60]: **I** (structureless pink-to-tan with irregular dots/globules located eccentrically) **II** (structureless pink-to-tan with peripheral vessels) **III** (structureless pink-to-tan) **IV** (network with raised, structureless, pink-to-tan areas) **V** (globular)
**MC1R**	70–90% of FM	Melanin production	-Red hair color phenotype [25,37] -Melanomas on trunk-arms [61] -Hypopigmented nevi [62] -Larger nevi and melanomas [63]	-Hypopigmentation [62] -Few dermoscopic structures [62] -Vascular pattern [62]
**PTEN**	NA	Tumor suppressor	-Loss of heterozygosity observed in approximately 30% of human melanomas [64] -Higher mutation frequency in pigmented nevi and melanomas of Xeroderma Pigmentosum patients [65]	-Multicomponent pattern associated with lower PTEN expression [66]
**TYR (OCA1)**	NA	Tyrosinase	-NA	-Hypopigmentation, vascular pattern [67]
**OCA2**	NA	Melanocyte-specific transporter protein	-NA	-Hypopigmentation, amelanotic melanoma [67]
**SLC45A2 (OCA 4)**	NA	Membrane-associated transporter protein	-NA	-Amelanotic melanoma [67] -Melanotic melanoma in case of increased expression of MITF [68]

FM (familial melanoma), SSM (superficial spreading melanoma), TNN (total nevi number), NA (not available).

**Table 2:** Mutation findings in our cohort of patients.

	Nucleotide	Protein Change	Mutation
**CDKN2A**	c.-34G>C c.-17G>T c.44 G>A c.71 G>C c.301G>T c.301G>C c.301G>T c.301G>T c.79G>T c.458-105A>G c.229A>G c.229A>G	- p.Gly6Val p.Trp15Ter p.Arg24Pro p.Gly101Trp p.Gly101Arg p.Gly101Trp p.Gly101Trp p.Glu27Ter - p.Thr77Ala p.Thr77Ala	intron variant missense nonsense missense missense missense missense missense nonsense intron variant missense missense
**MITF**	c.952 G>A c.952 G>A c.952 G>A	p.Glu318Lys p.Glu318Lys p.Glu318Lys	missense missense missense
**MC1R**	c.451C>T c.478C>T c.880G>C	p.Arg151Cys p.Arg160Trp p.Asp294His	missense missense missense
**BAP1**	c.368delG	p.Ser123Thrfs*64	nonsense
**TYR**	c.1217C>T c.1217C>T	p.Pro406Leu p.Pro406Leu	missense missense
**SLC45A**	c.1532C>A c.957C>A	p.Ala511Glu p.Tyr319Ter	missense nonsense
**OCA2**	c.1327G>A	p.Val443Ile	missense
**PTEN**	c.801G>A	p.Lys267Asn	missense

**Table 3:** Patient’s characteristics.

Mutation	N° pts	Sex	Age, Mean (Range)	Previous Melanomas, Mean (Range)	TNN, Mean (Range)	Body Site	Melanoma Type	Nevi Dermoscopy	Melanoma Dermoscopy
**CDKN2A**	12	7 F 5 M	43 (36–52)	6 (2–8)	21 (154–270)	Dorsum and upper limbs	MIS	-Pigmented reticular pattern -Hypopigmented	-Unstructured areas -Blotches -Atypical network -Pinkish areas -Atypical vascular structures
**MITF**	3	3 F	71 (62–80)	7 (5–9)	260 (130–390)	Upper limbs	2 MIS SSM 0.7 mm	-Pigmented reticular pattern	Non-specific pattern
**BAP1**	1	F	34	2 melanomas 1 BAPomas 1 AST	75	Dorsum	SSM 0.4 mm	-Hypomelanotic and amelanotic pattern	-Structureless pink-to-tan -Atypical vascular structures
**MC1R**	3	2F 1M	57 (40–72)	1	70 (50–90)	Dorsum	SSM 0.5 mm	-Pigmented reticular pattern	-Atypical, pigmented network
**PTEN**	1	M	56	1	150	Dorsum	SSM 0.8 mm	-Pigmented reticular pattern -Hypopigmented	-Atypical, pigmented network
**TYR (OCA1)**	2	2 F	45 (43–47)	2 (1–3)	159 (48–270)	Upper limbs, Trunk	SSM 0.5 mm MIS	-Reticular pattern -Hypopigmented	-Atypical, pigmented network
**OCA2**	1	M	58	3	300	Trunk	MIS	Pigmented reticular pattern	-Atypical pigmented network -Pinkish areas
**SLC45A2 (OCA 4)**	2	1 F 1 M	25 (26–29)	1 (1–1)	221 (172–270)	Trunk	SSM 0.5 mm, SSM 2.3 mm	Globular and reticular pattern	-Atypical pigmented network -Streaks

TNN (total nevi count), MIS (melanoma in situ), SSM (superficial spreading melanoma), BAPoma (BAP1-inactivated melanocytic tumor), AST (atypical Spitz nevus), NA (not available).

## Data Availability

The data can be shared up upon reasonable request to the corresponding author.
